# Discrete Illumination‐Based Compressed Ultrafast Photography for High‐Fidelity Dynamic Imaging

**DOI:** 10.1002/advs.202403854

**Published:** 2024-08-09

**Authors:** Jiali Yao, Zihan Guo, Dalong Qi, Shiyu Xu, Wenzhang Lin, Long Cheng, Chengzhi Jin, Yu He, Ning Xu, Zhen Pan, Jiayi Mao, Yunhua Yao, Lianzhong Deng, Yuecheng Shen, Heng Zhao, Zhenrong Sun, Shian Zhang

**Affiliations:** ^1^ State Key Laboratory of Precision Spectroscopy School of Physics and Electronic Science East China Normal University Shanghai 200241 China; ^2^ North Night Vision Technology Co. Ltd Kunming 650217 China; ^3^ Collaborative Innovation Center of Extreme Optics Shanxi University Taiyuan 030006 China; ^4^ Joint Research Center of Light Manipulation Science and Photonic Integrated Chip of East China Normal University and Shandong Normal University East China Normal University Shanghai 200241 China; ^5^ Present address: College of Science Shanghai Institute of Technology Shanghai 201418 China

**Keywords:** compressive sensing, compressed sensing, image reconstruction, pulse shaping, ultrafast imaging

## Abstract

Compressed ultrafast photography (CUP) can capture irreversible or difficult‐to‐repeat dynamic scenes at the imaging speed of more than one billion frames per second, which is obtained by compressive sensing‐based image reconstruction from a compressed 2D image through the discretization of detector pixels. However, an excessively high data compression ratio in CUP severely degrades the image reconstruction quality, thereby restricting its ability to observe ultrafast dynamic scenes with complex spatial structures. To address this issue, a discrete illumination‐based CUP (DI‐CUP) with high fidelity is reported. In DI‐CUP, the dynamic scenes are loaded into an ultrashort laser pulse train with controllable sub‐pulse number and time interval, thus the data compression ratio, as well as the overlap between adjacent frames, is greatly decreased and flexibly controlled through the discretization of dynamic scenes based on laser pulse train illumination, and high‐fidelity image reconstruction can be realized within the same observation time window. Furthermore, the superior performance of DI‐CUP is verified by observing femtosecond laser‐induced ablation dynamics and plasma channel evolution, which are hardly resolved in the spatial structures using conventional CUP. It is anticipated that DI‐CUP will be widely and dependably used in the real‐time observations of various ultrafast dynamics.

## Graphical abstract

By introducing ultrafast laser modulation into conventional receive‐only compressed ultrafast photography (CUP) as the illumination, discrete illumination‐based CUP with high imaging fidelity is achieved in a physical discretization way. The real‐time observations of femtosecond laser‐induced ablation dynamics and plasma channel evolution revolutionize the ability of CUP‐series techniques to observe ultrafast dynamic scenes with complex spatial structures.

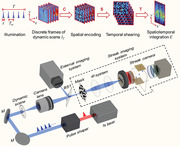

## 1 Introduction

Single‐shot ultrafast optical imaging (SUOI),^[^ ^1^, ^2^ ^]^ as a vibrant research field for real‐time visualization of unrepeatable or difficult‐to‐repeat ultrafast dynamic scenes at the imaging speed of more than one billion frames per second (fps), is a key technology for human comprehension across diverse transient physical, chemical and biological phenomena. Among SUOI technologies, compressed ultrafast photography (CUP)^[^ ^3^ ^]^ has become a pioneering receive‐only SUOI imaging technique, achieving an imaging speed of up to trillions of fps through the fusion of compressed sensing (CS) and streak imaging. The fundamental principle of CUP involves spatio‐temporal compression of a dynamic scene encoded by a spatially pseudo‐random binary pattern into a 2D observation image through streak imaging, followed by the utilization of a CS algorithm for reconstructing the original scene from the acquired 2D observation image.

Over the past decade, a series of SUOI techniques stemming from CUP have been developed, continuously achieving breakthroughs in imaging speed and imaging dimension. In the imaging speed aspect, the original CUP^[^ ^3^ ^]^ based on a streak camera for temporal shearing and ultrafast electro‐optical deflection imaging (UEODI)^[^ ^4^ ^]^ based on an electro‐optical deflector for temporal shearing have achieved imaging speeds in the 10^11^ fps range in a passive mode, which is restricted by the instrument response speeds of the deflectors. Alternatively, temporally chirped pulses or pulse trains are frequently used as the illumination source. Leveraging the one‐to‐one correspondence between wavelength and time, this method allows for the replacement of temporal shearing with spectral shearing to overcome the impediments posed by the response speed restrictions of temporal deflectors. Typically, compressed spectral‐temporal photography^[^ ^5^ ^]^ achieves an imaging speed of up to 3.85 × 10^12^ fps through the utilization of the spectral shearing method. Furthermore, the recently reported swept coded aperture real‐time femtophotography^[^ ^6^ ^]^ compresses and captures a dynamic scene carried by a temporally chirped pulse via spectral shearings in two opposite directions, achieving an extraordinary imaging speed of 1.56 × 10^14^ fps. Moreover, integrating the active illumination and spectral shearing approaches into the CUP modality can also boost the imaging speed. For example, compressed ultrafast spectral photography^[^ ^7^, ^8^ ^]^ elevates the imaging speed to a record of 2.19 × 10^14^ fps by introducing a temporally chirped pulse train as the illumination, as well as inserting an additional spectral shearing module orthogonal to the temporal shearing direction. For the imaging dimension elevation, various high‐dimensional spectral‐resolved,^[^ ^8^, ^9^ ^]^ volumetric‐resolved^[^ ^10^ ^]^ and polarization‐resolved^[^ ^11^, ^12^ ^]^ CUP techniques have been achieved. These advancements have enabled the successful applications of CUP in capturing flying photons,^[^ ^3^, ^13^ ^]^ measuring ultrafast optical fields,^[^ ^14^, ^15^, ^16^ ^]^ and monitoring optical chaos,^[^ ^17^ ^]^ optical rogue waves^[^ ^18^ ^]^ and ultrashort laser–matter interactions^[^ ^6^, ^12^, ^19^, ^20^, ^21^ ^]^ in real time.

Despite the outstanding imaging performance of CUP, the lossy encoding and spatio‐temporal compression sampling result in a severe negative impact on the reconstruction quality of the scene. As a typical computational optical imaging modality, a reconstruction algorithm with better performance can directly improve the imaging quality of CUP. Consequently, as alternatives to the traditional iterative algorithms initially applied to CUP, a series of end‐to‐end deep learning algorithms,^[^ ^22^, ^23^, ^24^ ^]^ as well as plug‐and‐play algorithms combining the traditional iterative framework with deep denoising,^[^ ^16^, ^25^, ^26^ ^]^ have been continuously developed for high quality reconstruction of scenes. More importantly, the image acquisition quality of the hardware configuration plays a decisive role in the fidelity of a CUP system. In recent years, researchers have made several improvements to the hardware configuration of CUP in addition to developing high‐performance CS reconstruction algorithms. Zhu et al. improved the spatial resolution, contrast, and general quality of the reconstructed scene by adding an external camera into the CUP system to acquire a time‐unsheared image, which can provide spatial and intensity constraints as additional prior information.^[^ ^27^ ^]^ Yang et al. utilized a genetic algorithm to obtain the optimal encoding pattern in CUP, which can configure the best sensing matrix for undersampling.^[^ ^28^ ^]^ Lossless‐encoding CUP^[^ ^13^ ^]^ and multichannel‐coupled CUP^[^ ^29^, ^30^ ^]^ provide more spatial details of the scenes by using multiple imaging acquisition channels with different encoding patterns to increase the sampling rate. Alternatively, compressed optical‐streaking ultra‐high‐speed photography^[^ ^31^ ^]^ based on a galvanometer deflector and UEODI^[^ ^4^ ^]^ based on an electro‐optical deflector have significantly improved the imaging quality with an all‐optical streaking solution to avoid the image blurring problem caused by the space‐charge effect in streak camera.

As mentioned above, the current emphasis in hardware systems lies in improving the spatial encoding and temporal deflector modules, overlooking the influence of the compressive sampling process on scene reconstruction. Currently, CUP typically captures a time‐continuously evolving ultrafast dynamic scene and performs spatio‐temporal discretization recovery of the scene based on detector pixels during the reverse reconstruction process, typically involving the reconstruction of a few hundred of image frames for temporal imaging. Moreover, the increases in imaging dimension and speed lead to an approximately an order of magnitude increase in the number of reconstructed frames. Such a large data compression ratio imposes greater pressure on the scene reconstruction compared to the lossy encoding. Therefore, we developed a discrete illumination‐based CUP (DI‐CUP) considering the spatio‐temporal compression sampling process of CUP. By temporally shaping a femtosecond laser pulse into a pulse train to provide discrete ultrafast illumination for dynamic scenes, DI‐CUP can autonomously reduce the data compression ratio and the overlap between adjacent frames, thereby realizing high‐fidelity reconstruction of the scene. Here, we measured a static resolution test target using DI‐CUP and conventional CUP, respectively, to demonstrate the significant advantages of DI‐CUP in enhancing spatial resolution. In addition, the ultrafast evolutions of femtosecond laser‐induced silicon ablation and air plasma channel were measured by DI‐CUP and compared with those obtained by the pump‐probe microscopy measurements, demonstrating the potential of DI‐CUP in recording ultrafast dynamics with complex spatial structures.

## 2 Experimental Section

### 2.1 Theoretical Model

**Figure 1:**
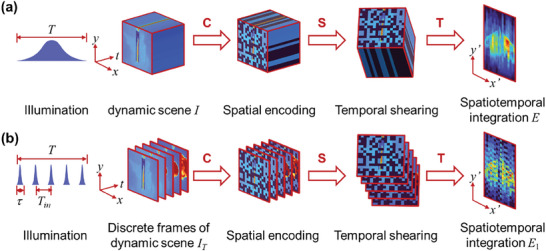
Data acquisitions of CUP (a) and DI‐CUP (b). Here, *t*: time; *x*, *y*: spatial coordinates of the dynamical scene; *x*′, *y*′: spatial coordinates at the streak camera.

In conventional CUP systems, a laser pulse with a duration comparable to the timescale of an ultrafast event is typically used as the illumination light to form a time‐continuously evolving dynamic scene *I*(*x*, *y*, *t*). Subsequently, CUP compresses this dynamic scene into a 2D observation image *E*(*x*′, *y*′) through spatial pseudo‐random coding **C** from a spatial mask, temporal shearing **S** from a sweeping voltage in the streak camera, and spatio‐temporal integration **T** from a planar detector, as shown in **Figure** **1**a. The forward data acquisition process can be mathematically formulated as:^[^ ^3^ ^]^

(1) $$E\left(x^{\prime },y^{\prime }\right)=TSCI\left(x,y,t\right)$$

For reverse reconstruction, CUP relies on a CS algorithm to find the optimal estimation I* of *I* by solving an objective function *f*(*I*) as follows:^[^ ^32^, ^33^, ^34^ ^]^

(2) $$I^{*}=\underset{I}{\arg\min}f\left(I\right)=\underset{I}{\arg\min}\frac{1}{2}{\left\|E-\mathrm{TSC}I\right\|}_{2}^{2}+\lambda \Phi \left(I\right)$$

where ||•||_2_ is the *l*_2_ norm, ${∥E-\mathrm{TSC}I∥}_{2}^{2}$ is the fidelity term, Φ(*I*) is the regularization term, and *λ* is the regularization parameter to balance the two terms. Equation (2) is usually solved using iterative algorithms, and the existence of different solvers depends on the forms of Φ(*I*).^[^ ^35^ ^]^

In the reconstruction process, the 2D observation image *E*(*x*′, *y*′) is discretized into a data‐matrix based on the detector pixels, and the adjacent frames of the scene are considered to be deflected downward by one pixel in the temporal shearing **S**. Therefore, for a dynamic scene with a duration of *T*, the total number of reconstructed frames *F* can be derived as:

(3) $$F_{CUP}=\mathrm{Floor}\left(v*T/\Delta y^{\prime }\right)+1$$

where Δ*y*′ is the detector's pixel size along the temporal shearing direction, *v* is the temporal shearing velocity, and Floor(·) indicates rounding down. The temporal resolution *r* is proportional to the encoding pixel size and is determined by the certain time interval corresponding to each encoding pixel at a specific shearing velocity, which can be expressed as:^[^ ^3^, ^11^ ^]^

(4) $$r=\Delta d^{\prime }/v$$

where Δ*d*′ is the encoding pixel size along the temporal shearing direction. If the mask pixels are mapped 1:1 to the detector pixels, that is, Δ*d*′ = Δ*y*′, and perfectly registered, combining Equations (3) and (4) yields

(5) $$F_{CUP}=\mathrm{Floor}\left(T/r\right)+1$$

As can be seen from Equation (5), the higher temporal resolution represents an increased number of image frames for reconstruction. However, excessive data compression resulting from the restoration of abundant original data through minimal observations can seriously degrade the spatial fidelity of the reconstructed images. Therefore, there is a constraint between temporal resolution and reconstruction quality in CUP.

To solve this problem, we designed the DI‐CUP imaging model, whose forward data acquisition process is shown in Figure 1b. Instead of using a single pulse, a series of temporally separated ultrashort pulses, with the time interval between adjacent pulses much larger than the duration of the pulse, are used as the illumination source to acquire the time‐slice sub‐scene sequence of an ultrafast event, and the duration of the whole pulse train is comparable to that of the single illumination pulse shown in Figure 1a. The timestamp for each sub‐pulse can be expressed as *t*(*n*, *τ*) = *nT_in_* + *t*_1_, *t*_1_∈[0, *τ*], *n* is the sub‐pulse index (*n* = 0, 1, 2, 3…), *T_in_* is the time interval between adjacent sub‐pulses, and *τ* is the duration of each sub‐pulse. Consequently, the transmitted light carrying the dynamic scene has the form of *I_T_* (*x*, *y*, *t*(*n*,*τ*)), and the forward acquisition equation of DI‐CUP can be written as:

(6) $$E_{1}\left(x^{\prime },y^{\prime }\right)=TSCI_{T}\left(x,y,t\left(n,\tau \right)\right)=TSCDI\left(x,y,t\right)$$

where *I_T_* (*x*, *y*, *t*(*n*,*τ*)) = **D** *I*(*x*, *y*, *t*), and **D** is the temporal discretization of the time‐continuously evolving dynamic scene *I*(*x*, *y*, *t*) by the probe pulse train. In DI‐CUP, when the streak camera operates at a higher shearing velocity (higher temporal resolution), the sub‐scenes recorded by neighboring sub‐pulses are deflected to separate several detector pixels that are free of photons compared to CUP. In this case, the number of reconstructed frames is determined by:

(7) $$F_{DI-CUP}=N\cdot \left(\mathrm{Floor}\left(v\tau /\Delta y^{\prime }\right)+1\right)=N\cdot \mathrm{Floor}\left(\tau /r\right)+N$$

where *N* is the number of sub‐pulses. When *τ < *Δ*y*′/*v*, the sub‐scene recorded by a sub‐pulse is considered to be an image frame that is not sheared on the detector, and thus Equation (7) changes to *F_DI‐CUP_*  = *N*, that is, the number of reconstructed frames is a constant. Since the pulse train with *τ* ≪ *T*_in_ is used, *F*_DI‐CUP_ ≪ *F*_CUP_, and thus the data compression ratio is significantly reduced relative to that of CUP, which contributes to the improved image reconstruction quality at high temporal resolution.

### 2.2 Experimental System

**Figure 2:**
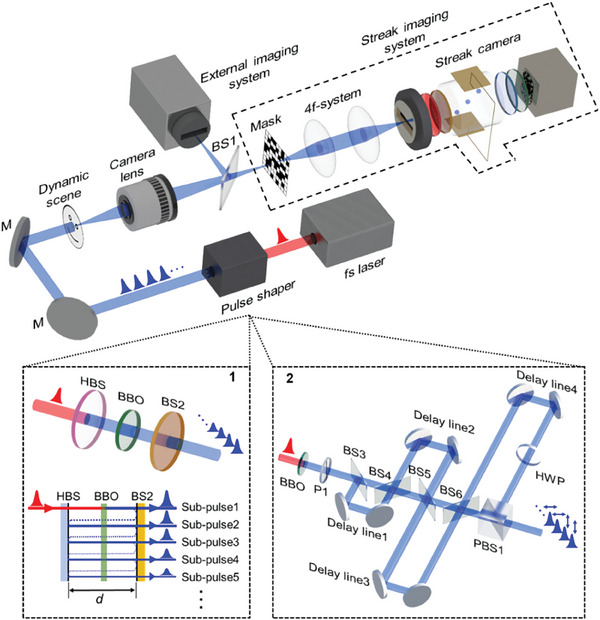
Experimental arrangement of DI‐CUP. Inset 1: Pulse shaper based on FP‐cavity structure; Inset 2: Pulse shaper based on cascaded beam splitting structure. BS, beam splitter; L, lens; M, mirror; P, polarizer; HBS, harmonic beam splitter; BBO, β‐BaB_2_O_4_; HWP, half‐wave plate; PBS, polarized beam splitter.

The schematic diagram of the DI‐CUP is shown in **Figure** **2**. A Ti: Sapphire femtosecond laser amplifier generates a laser pulse with a central wavelength of 800 nm and a pulse width of 35 fs. The pulse is converted into a pulse train with a central wavelength of 400 nm by a pulse shaper, and the pulse train is then directed onto a dynamic scene as successive “flashes”. The photons are collected by a camera lens to form an intermediate scene. Subsequently, a beam splitter (BS1) separates the intermediate scene into two components. The transmitted component is imaged on a mask with a pseudo‐random binary pattern and then the encoded scene passes through an optical 4f system (magnification 1×) consisting of two lenses before entering the streak camera to form the time‐sheared view. The reflected component is captured by an external imaging system to generate the time‐unsheared view, providing an additional prior for subsequent scene reconstruction. In the calibration process, the image distortion between the two views is calibrated based on the standard camera calibration matrix.^[^ ^8^, ^11^ ^]^

Here, two pulse shaper designs were employed to generate distinct pulse trains. One design utilizes a compact Fabry–Perot (FP) cavity structure to produce a pulse train with diminishing sub‐pulse intensities, as shown in inset 1 of Figure 2. The FP‐cavity structure comprises a harmonic beam splitter (HBS) with a high reflection of 400 nm and a high transmission of 800 nm, as well as a conventional broadband beam splitter (BS2) with a reflectance to transmittance ratio of 9:1. A *β*‐BaB_2_O_4_ (BBO) crystal is inserted into the FP‐cavity for frequency doubling. Here, the 400‐nm laser pulse generated by frequency doubling undergoes continuous reflection within the FP cavity, transmitting a small portion each time it encounters BS2. Consequently, a series of discrete sub‐pulses with diminishing intensities are provided. In this case, the time interval between each adjacent sub‐pulses depends on the cavity length *d* of the FP cavity, can be easily and flexibly adjusted from a few picoseconds to nanoseconds. The other design employs a cascaded beam‐splitting structure to create a pulse train with equal sub‐pulse intensities, as shown in inset 2 of Figure 2. First, the 400‐nm laser pulse generated by a BBO crystal is transformed into the *s*‐polarized state through a polarizer (P1). Subsequently, the *s*‐polarized pulse enters a cascaded beam‐splitting structure consisting of BS3‐6 (split ratio 5:5) and delay lines 1–4, resulting in the generation of 16 sub‐pulses, with 8 along each of the transmission and reflection directions of BS6. Finally, the reflected and transmitted sub‐pulses are collinearly combined by a half‐wave plate (HWP) and a polarized beam splitter cube (PBS1), and thus, the 16 sub‐pulses with equal intensities compose into a pulse train for illumination. In this configuration, the first 8 sub‐pulses are *s*‐polarized and the next 8 sub‐pulses are *p*‐polarized, and the time interval between adjacent sub‐pulses can be flexibly adjusted by changing the length of the delay line on the corresponding branch. The overall system is synchronized with a time delay generator.^[^ ^36^ ^]^ See Note S1 (Supporting Information) for details on the optical components.

## 3 Results

### 3.1 Theoretical Comparison of the Image Qualities between DI‐CUP and CUP

In order to prove that the reduction of the data compression ratio can effectively improve the quality of image reconstruction, a series of numerical simulations were first performed. Here, five dynamic scenes (Welding, Filament, Gas, Detonators, and Water) captured by high‐speed cameras were sequentially spatially coded, temporally sheared, and spatiotemporally integrated to simulate the forward acquisition process. Each scene contains 15 images of size 256 × 256. For spatial encoding, each image frame of the scene was encoded by an identical pseudo‐random binary pattern with the pixel size matching that of the detector. For temporal shearing, these encoded images were shifted sequentially with each frame moved down by ten pixels relative to the previous one. For spatiotemporal integration, the intensities of the encoded and sheared image frames at each pixel were superimposed onto the detector. As a result, the 2D observation image size for each scene was 396 × 256. Subsequently, based on the imaging principles of DI‐CUP and CUP, two different high‐performance CS algorithms including the fast and flexible denoising convolutional neural network (FFDNet)‐based plug‐and‐play (PnP) framework algorithm (PnP‐FFDNet)^[^ ^16^ ^]^ and the total variation (TV) and cascaded denoisers (CD)‐based PnP framework algorithm (PnP‐TV‐CD)^[^ ^26^ ^]^ were respectively used to recover the original scenes from the observation images for comparison. For CUP, the size of each observation image along the *y*′‐axis is considered to be obtained by sequentially deflecting each frame downward by one pixel with respect to the previous frame, thus a total of 141 image frames were reconstructed for each scene. On the contrary, in DI‐CUP, the number of deflected pixels in adjacent frames is considered to be 10, thus a total of 15 images were reconstructed, meaning that the data compression ratio is 1/9.4 of that in CUP.

**Figure 3:**
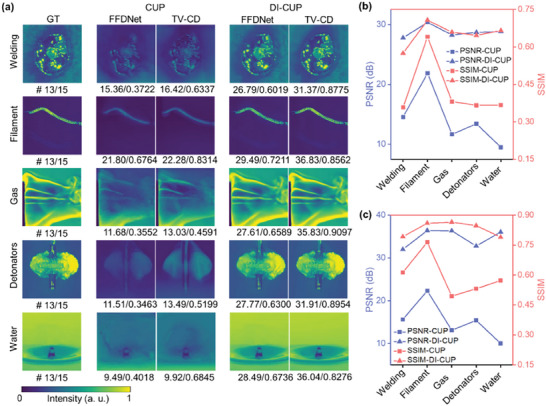
Theoretical comparison of the image qualities between DI‐CUP and CUP for five dynamic scenes (a), together with averaged PSNR in dB and SSIM values through FFDNet (b) and TV‐CD (c) based image reconstruction.

**Figure** **3**a shows the 13th freeze‐frame in 15 ground truth (GT) frames of the five scenes and the reconstruction results of both algorithms for CUP and DI‐CUP. Here, the peak signal‐to‐noise ratio (PSNR) values in dB and structural similarity (SSIM) values are labeled below the image. It can be seen that the reconstruction results from DI‐CUP exhibit significantly higher PSNR and SSIM values compared to CUP. Additionally, the PSNR and SSIM values obtained by the TV‐CD algorithm surpass those of the FFDNet algorithm. Overall, all the reconstructed images from CUP appear blurry, featuring only rough outlines and lacking internal details, for example, the spiral structure inside the filament could not be recovered. Even the reconstruction results contain serious errors, for example, the explosion center of the detonators still looks intact. In addition, the spatial intensities of the reconstructed images are notably lower than those of GTs. In contrast, the reconstruction results from DI‐CUP are markedly superior, featuring clear boundaries and high contrast in the reconstructed images. Not only the correct spatial intensity information is obtained, but also almost all complex detail information is preserved. In order to show their differences more concretely, the averaged PSNR and SSIM values of all reconstructed image frames of five scenes obtained by the two algorithms were calculated, and the results are shown in Figure 3b,c, respectively. It can be obviously seen that DI‐CUP outperforms CUP significantly in terms of image reconstruction quality parameters for both algorithms, with PSNR ≈10 dB higher and SSIM ≈0.15 higher, indicating that the scenes recorded by DI‐CUP more closely resemble GTs. Moreover, to comprehensively explore the imaging performance of DI‐CUP under different conditions, simulations with various spatial shift intervals between adjacent frames for reconstruction and non‐uniform intensities of sub‐pulses were performed, respectively. Figure S1 and Note S2 (Supporting Information), provide the results with various spatial shift intervals, which indicate that a larger spatial shift interval under the same compression ratio is beneficial to improve the reconstructed image quality by decreasing the spatial overlap between adjacent frames. Figures S2,S3 and Note S3 (Supporting Information), provide the results with non‐uniform intensities, which demonstrate that images can be reliably reconstructed within an intensity fluctuation of 20%. Optimally, the intensity fluctuation should be pre‐calibrated for further eliminating its effect on image reconstruction when the fluctuation cannot be ignored.

### 3.2 Experimental Comparison of the Spatial Resolutions between DI‐CUP and CUP

**Figure 4:**
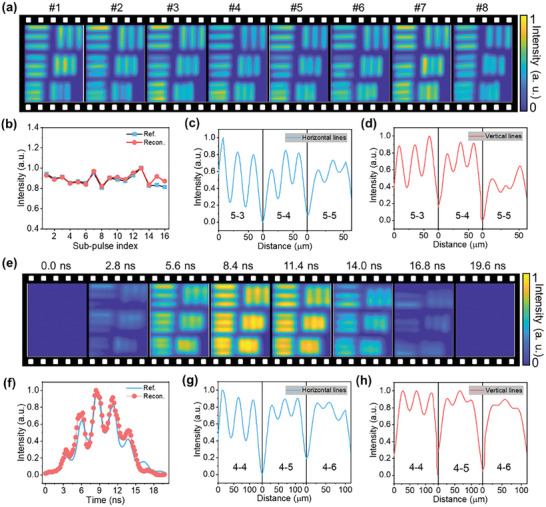
Experimental comparison of the spatial resolutions between DI‐CUP and CUP. a,e) Representative frames of the reconstruction results by DI‐CUP and CUP; b,f) Extracted temporal intensities from the reconstruction results of DI‐CUP and CUP; c,d) Integrated intensities of horizontal and vertical lines in #1 frame of (a); g,h) The same as (c) and (d) but in the frame at 8.4 ns of (e).

Furthermore, it is critical to demonstrate the spatial resolution improvement of DI‐CUP based on an actual experimental scene. Here, a static 1951 USFA resolution test target was measured using DI‐CUP and CUP, respectively. In DI‐CUP, we employed the second pulse shaper design, which can generate 16 sub‐pulses with uniform intensities. The time interval between adjacent sub‐pulses was set to ≈500 ps, corresponding to a duration of 7.5 ns for the pulse train. The dynamic scene was spatially encoded by a transmissive random mask with a pixel size of 80 μm, and the observation image was captured by a streak camera (Hamamatsu C7700) at the 20‐ns sweeping time window, which has a temporal shearing velocity of 3.25 × 10^5^ m s^−1^. The original scene was reconstructed using the TV‐CD algorithm, incorporating the space‐intensity constraint in the reconstruction process.^[^ ^27^ ^]^ The representative frames of reconstruction are shown in **Figure** **4**a. Each reconstructed image contains three sets of horizontal and vertical stripe patterns, representing elements 5‐3, 5‐4, and 5‐5 in the resolution test target, respectively. The number at the top of each reconstructed image represents the serial number of the sub‐pulse. It can be seen that the spatial intensities of the images recorded by the different sub‐pulses show minimal differences between each other. For quantitative evaluation, we extracted the overall intensities of all 16 reconstructed images by integrating over all pixel values and compared them with the sub‐pulse intensities measured by the streak camera in 1D streak mode.^[^ ^36^ ^]^ The results are shown in Figure 4b, where the reconstructed sub‐pulse intensity profile is highly consistent with the 1D measurement of the streak camera, thereby validating the accuracy of the reconstruction results. To further illustrate the image reconstruction quality, the intensities of these elements with the horizontal (vertical) lines in the first reconstructed image are integrated along the horizontal (vertical) direction, as shown in Figure 4c,d, respectively. In elements 5–3 and 5–4, the horizontal and vertical line structures can be easily distinguished. While in elements 5–5, although the horizontal line structure can still be recognized completely, the vertical line structure cannot. Therefore, the spatial resolution of DI‐CUP can be determined as ≈45.3 lp mm^−1^ for elements 5–4.

In CUP, a single pulse with a duration of 20 ns was employed as the illumination light and the observation image was captured at the 50‐ns sweeping time window of the streak camera, which has a temporal shearing velocity of 1.3 × 10^5^ m s^−1^. Since the duration of the illumination pulse is ≈2.5 times that of the illumination pulse train in DI‐CUP, the temporal shearing velocity was reduced to 1/2.5 of that in DI‐CUP to maintain a similar size of the 2D observation image along *y*' direction as in DI‐CUP. Here, a total of 392 image frames were reconstructed and eight representative frames are shown in Figure 4e. The three sets of horizontal and vertical stripe patterns in the reconstructed image are elements 4–4, 4–5, and 4–6 in the resolution test target, respectively. The entire spatio‐temporal evolution of the laser pulse, from its appearance to disappearance, is clearly observable in Figure 4e. Similarly, the evolution of the overall intensity of the laser pulse over time from all the reconstructed images was extracted, with the direct measurement result of the streak camera in 1D mode serving as the reference. As shown in Figure 4f, the two profiles exhibit good agreement. Moreover, the reconstructed image at 8.4 ns is selected and the integral intensities of the horizontal and vertical lines are calculated, and the results are shown in Figure 4g,h, respectively. It is seen that the horizontal and vertical line structures in elements 4–4 and 4–5 can be distinguished, but those in 4–6 cannot, indicating that the spatial resolution of CUP is 25.4 lp mm^−1^ for elements 4–5. Therefore, the experimental results of the resolution test target show that DI‐CUP achieves over a 78% spatial resolution improvement by significantly reducing the data compression ratio, proving the great advantage of DI‐CUP in realizing high‐fidelity reconstruction of a dynamic scene.

### 3.3 Experimental Validation of the Superior Performance of DI‐CUP

**Figure 5:**
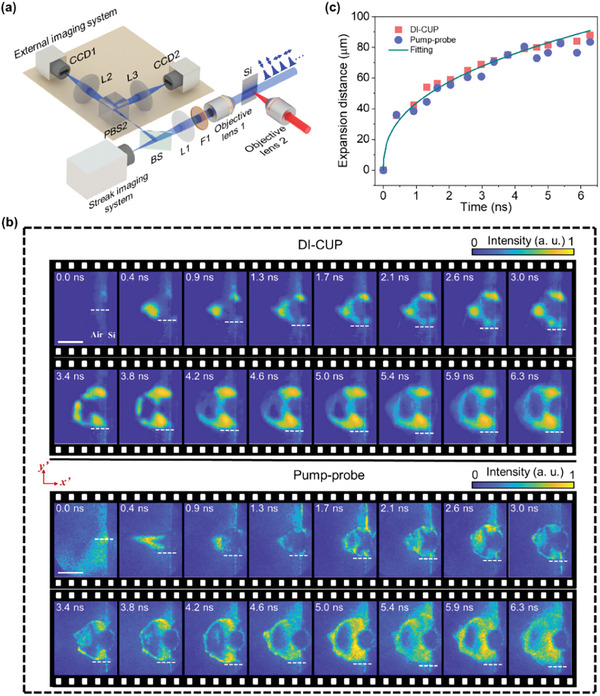
Real‐time observation of ultrafast laser ablation on silicon surface by DI‐CUP. a) Experimental setup; b) Reconstructed results by DI‐CUP, associated with the results by pump‐probe method for comparison; c) Radial expansion distances of the shockwave measured by DI‐CUP and pump‐probe method, together with the fitting curve. Scale bar: 100 μm.

To show the broad utility of DI‐CUP, we used it to monitor the ultrafast dynamics of a femtosecond laser ablation on a silicon surface based on the shadowgraph method, which is commonly applied to study the physical mechanisms of laser–matter interactions. As shown in **Figure** **5**a, the output pulse of the Ti: Sapphire laser amplifier is divided into two parts, one of which is focused by objective lens 2 as the pump light to ablate a silicon target. Here, the silicon target is placed near the beam waist of the focused spot, and the laser fluence is estimated to be 60 J cm^−2^, which is far beyond the damage threshold of silicon. The other is converted into a pulse train as a probe light by the cascaded beam‐splitting‐based pulse shaper, which illuminates the target along a direction perpendicular to the pump light. Here, the time interval between adjacent sub‐pulses is adjusted to ≈400 ps, so that the pulse train covers a total observation window of 6 ns. Subsequently, the ablation scene is relayed by the objective lens 1 (10×, NA 0.25) and a lens (L1, *f *= 400 mm) into the streak imaging system at the 20‐ns sweeping time window to acquire a time‐sheared view, and the time‐unsheared view is acquired by the external imaging system. Considering that the first eight sub‐pulses of the illumination pulse train are *s*‐polarized and the last eight are *p*‐polarized, we added an additional polarization beam splitter cube (PBS2) in the external imaging system to separate *p*‐polarized sub‐pulses from *s*‐polarized sub‐pulses and acquired two time‐unsheared views using two CCDs. In addition, a 400 nm bandpass filter (F1) together with some neutral density filters is used to prevent the residual 800 nm component in the probe beam and the fluorescence generated during the ablation from entering the imaging systems. After the data acquisition process, the TV‐CD algorithm was used to reconstruct the original ablation scene from the time‐sheared image, and space‐intensity constraint was applied to the reconstructed images in iterations based on these two images in the time‐unsheared views. The reconstructed image frames captured by DI‐CUP are shown at the top of Figure 5b. It can be seen that an opaque region appears on the target surface at 0.4 ns after the pump pulse impacted the target, with a high‐intensity conical structure attaching in front of the opaque region. Due to the rapid deposition of intense laser energy, the atoms on the target surface are ionized, forming a dense plasma that shields the illumination light and result in the generation of the opaque region.^[^ ^37^, ^38^ ^]^ Since the target is placed at the beam waist of the laser focal spot, the high‐intensity conical region is regarded as the leading edge of the plasma channel formed by the laser‐ionized air. With the lapse of time, the high‐temperature plasma expands outward, compressing the ambient air and forming a bright shockwave. Here, from 1.3 to 3.0 ns, there is a bulge existed on the shockwave front, which is attributed to the intense laser‐induced air breakdown, offering a fast channel for the propagation of shockwave due to the lower localized pressure.^[^ ^39^, ^40^ ^]^ Subsequently, from 3.4 to 6.3 ns, that is, the end of the observation time window, as the dense plasma expands and becomes thinner, probe light can gradually penetrate the plasma. During this stage, the opaque region near the target surface is mainly caused by the fragments, particles, and/or droplets ejected from the target.^[^ ^41^ ^]^ Simultaneously, another sizeable dark area can be observed behind the shockwave front, which may originate from the partial reflection caused by the collision of vapors in the ablation plume with the surrounding air, resulting in local density and pressure to rise.^[^ ^42^ ^]^ In order to verify the accuracy of DI‐CUP, we used a conventional pump‐probe microscopy to record the ablation scene through multiple exposures, and the results are shown at the bottom of Figure 5b. It can be seen that the morphological evolution of the ablation scene captured over time by pump‐probe is highly consistent with that captured by DI‐CUP. Limited by the spatial resolution of the current DI‐CUP system, the fan‐shaped structure extending from the plasma at 1.7–3.0 ns in the pump‐probe measurements is not clearly observed, which is driven by a phase explosion.^[^ ^42^ ^]^ Furthermore, we extracted the evolution of the radial (*y*′‐axis) expansion distance of the shockwave over time from the reconstruction images of DI‐CUP (the shockwave boundaries are shown as the white dashed lines in Figure 5b), and fitted the distance data based on the Sedov–Taylor theory, as shown in Figure 5c. According to Sedov–Taylor theory^[^ ^43^ ^]^ for a hemispherical shockwave, the relation between the radius *R* of the shockwave front and the time *t* may be expressed as *R  *∝  *Bt* ^2/5^, where *B* is a constant that depends on the energy to drive the shockwave and the density of undisturbed air. It is clear that the shockwave expansion radius measured by DI‐CUP agrees well with the Sedov–Taylor theoretical fitting, with an average expansion velocity of ≈14 km s^−1^. In addition, the time evolution of the expansion radius from the measurements of the pump‐probe method was also extracted and shown in Figure 5c for comparison. The overall trend of the evolution of the expansion radius over time measured by the pump‐probe is similar to that of DI‐CUP, except for the unreasonable phenomenon of radius shrinkage in certain frames, such as 4.6 and 5.9 ns, which most likely results from the instability of the pump pulses, and can be avoided by DI‐CUP as a single‐shot imaging technique.

**Figure 6:**
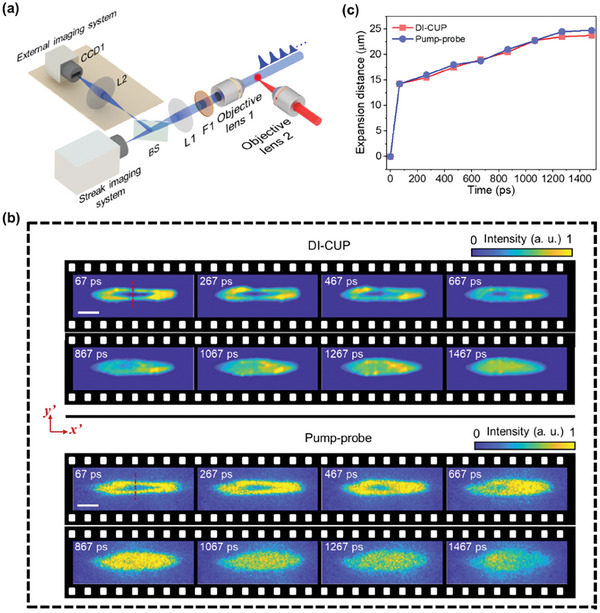
Real‐time observation of ultrafast laser‐induced plasma channel evolution in air by DI‐CUP. a) Experimental setup; b) Reconstructed results by DI‐CUP, associated with the results by pump‐probe method for comparison; c) Radial expansion distances of the plasma channel measured by DI‐CUP and pump‐probe method. Scale bar: 50 μm.

To highlight DI‐CUP's single‐shot movie‐shooting capability, we used the DI‐CUP to visualize the femtosecond laser‐induced plasma channel evolution in the air at a higher imaging speed. As shown in **Figure** **6**a, the objective lens 2 focuses the pump laser in air with a laser fluence of ≈60 J cm^−2^ at the focused spot, which exceeds the air breakdown threshold. A pulse shaper based on the FP cavity structure generates a pulse train with diminishing sub‐pulse intensities as the illumination light irradiating the focused spot, and the time interval between adjacent sub‐pulses is adjusted to 200 ps. Figure S4 and Note S4 (Supporting Information), depict the intensity distribution of sub‐pulses in 1D streak mode. Subsequently, the streak imaging system at the 5‐ns sweeping time window receives photons collected by objective lens 1 and the lens (L1). Due to the identical polarization of all sub‐pulses, a time‐unsheared image is acquired by only CCD1 in the external imaging system. Similarly, the 400 nm band‐pass filter (F1) is positioned behind objective lens 1 to eliminate both pump light and fluorescence. After the data acquisition process, the TV‐CD algorithm was used to reconstruct the scene, and a total of 8 image frames were reconstructed, as shown at the top of Figure 6b. At 67 ps, it can be observed that the laser‐induced plasma channel has formed as an elliptical narrow region. As time elapses, the plasma channel expands and widens along the *y*′‐axis. It is worth noting that at 67 ps, there is a clear oval‐like hollow structure inside the plasma channel, which then gradually diminishes until it completely disappears at 1067 ps. The hollow structure was previously studied by Li et al. and was thought to arise from the interplay between the diffraction induced by the breakdown plasma and the refocusing induced by the Kerr effect under the condition of femtosecond laser tight focusing.^[^ ^44^ ^]^ Similarly, the pump‐probe method was used to record the scene to verify the accuracy of DI‐CUP, and the results are shown at the bottom of Figure 6b. The plasma channel morphology and the time evolution of the hollow structure observed by DI‐CUP are in high agreement with those observed by the pump‐probe. In addition, the time evolution of the expansion distance of the plasma channel along the red dashed line was also extracted for comparison, as shown in Figure 6c. Obviously, the expansion distances measured by the two techniques match well, with an average expansion velocity of ≈16 km s^−1^, proving the reliability of DI‐CUP for real‐time visualization.

## 4 Discussion

In summary, we have developed a discrete illumination‐based compressed ultrafast photography (DI‐CUP) technique. Compared with CUP, DI‐CUP converts an ultrashort pulse into a series of discrete sub‐pulses by using temporal pulse shaping. This approach offers discrete ultrafast illumination for dynamic scenes, thereby significantly reducing the data compression ratio and the overlap between adjacent frames in the forward acquisition process. Both theoretical and experimental results show that DI‐CUP can obtain superior spatial resolution and excellent imaging quality. In addition, we have also used DI‐CUP to record the ultrafast evolutions of femtosecond laser‐induced silicon ablation and air plasma channel, demonstrating the high fidelity and reliability of DI‐CUP in recording ultrafast dynamics with complex spatial structures. In the future, the number of sub‐pulses will become more flexible and controllable with the aid of more advanced hardware‐based pulse shapers, such as acousto–optics programmable dispersive filter,^[^ ^45^ ^]^ free‐space angular‐chirp‐enhanced delay,^[^ ^46^ ^]^ and spectrum shuttle,^[^ ^47^ ^]^ which will ensure that DI‐CUP achieves the optimal trade‐off between the imaging quality and the number of imaging frames in the observation of different dynamic scenes. In software, the constantly updated CS algorithm based on deep learning can also effectively reduce the reconstruction artifacts caused by the severe distortion of the scene along the *y*′‐axis at high shearing velocity. There is no doubt that the imaging performance of DI‐CUP will be further improved. What's more, by leveraging the multiple scanning speed of the streak camera, DI‐CUP can span a large time range of picoseconds to milliseconds. Moreover, by adjusting the magnification of the input optics, DI‐CUP can also be scaled in the spatial range of sub‐micrometers to centimeters. As a result, DI‐CUP can monitor the complex reaction processes of various targets including solids, fluids, plasmas, and biological cells and tissues. We believe that the DI‐CUP will play an impactful role in diverse ultrafast dynamic real‐time observations, and accelerate unexpected discoveries in science and industry.

## Conflict of Interest

The authors declare no conflict of interest.

## Supporting information

Supporting Information

## Data Availability

The data that support the findings of this study are available from the corresponding author upon reasonable request.
