# Supplementary material for: Discrete Illumination‐Based Compressed Ultrafast Photography for High‐Fidelity Dynamic Imaging
Source: Adv Sci (Weinh). 2024 Aug 9;11(41):2403854. doi: 10.1002/advs.202403854 (PMC11538675; doi:10.1002/advs.202403854)
Supplement: Supplementary file 1 — Supporting Information [file ADVS-11-2403854-s001.pdf]

ADVANCED SCIENCE
Open Access

Supporting Information

for *Adv. Sci.*, DOI 10.1002/advs.202403854

Discrete Illumination-Based Compressed Ultrafast Photography for High-Fidelity Dynamic Imaging

Jiali Yao, Zihan Guo, Dalong Qi, Shiyu Xu, Wenzhang Lin, Long Cheng, Chengzhi Jin, Yu He, Ning Xu, Zhen Pan, Jiayi Mao, Yunhua Yao, Lianzhong Deng, Yuecheng Shen, Heng Zhao, Zhenrong Sun and Shian Zhang**

Supporting Information for

Discrete illumination-based compressed ultrafast photography for high-fidelity dynamic imaging

Jiali Yao[1,5,#], Zihan Guo[1,#], Dalong Qi[1,*], Shiyu Xu[2], Wenzhang Lin[1], Long Cheng[1], Chengzhi Jin[1], Yu He[1], Ning Xu[1], Zhen Pan[1], Jiayi Mao[1], Yunhua Yao[1], Lianzhong Deng[1], Yuecheng Shen[1], Heng Zhao[2], Zhenrong Sun[1], Shian Zhang[1,3,4,*]

[1]*State Key Laboratory of Precision Spectroscopy, School of Physics and Electronic Science, East China Normal University, Shanghai 200241, China.*

[2]*North Night Vision Technology Co. Ltd, Kunming, 650217, China.*

[3]*Collaborative Innovation Center of Extreme Optics, Shanxi University, Taiyuan 030006, China.*

[4]*Joint Research Center of Light Manipulation Science and Photonic Integrated Chip of East China Normal University and Shandong Normal University, East China Normal University, Shanghai 200241, China.*

[5]*Present address: College of Science, Shanghai Institute of Technology, Shanghai 201418, China.*

[#]*The authors contributed equally to this work.*

[*]*Corresponding authors: dlqi@lps.ecnu.edu.cn; sazhang@phy.ecnu.edu.cn*

Supporting Note 1 **DI-CUP system details**

1.1 Equipment

In Fig. 2, two different structures of pulse shapers were used in the experiments. The FP cavity-based pulse shaper in Inset 1 consists of a harmonic beam splitter (HBS, Newport, 10Q20UF.HR40), a BBO crystal (MT Optics, I-type) and a beam splitter (BS2, Boxin Photoelectric, customized). The cascaded beam-splitting-based pulse shaper in Inset 2 consists of a BBO crystal (MT Optics, I-type), a polarizer (P1, Thorlabs, WP25L-VIS), a polarized beam splitter (PBS1, Lbtek, MPBS24-405), a half-wave plate (HWP, Lbtek, HWP20-405B), several beam splitters (BS3-BS6, Lbtek, customized) and several mirrors (Thorlabs, BB1-E02). The streak imaging system consists of a spatial mask (PointVision customized), a 4f system (Thorlabs, MAP10100100-A) and a streak camera (Hamamatsu, C7700). A femtosecond laser (Coherent, Astrella USP) was used to generate the femtosecond pulse with the central wavelength of 800 nm. The laser, streak camera and external CCD are precisely synchronized by a digital delay generator (Stanford Research Systems, DG645).

1.2 Ultrafast imaging configuration

In Fig. 5(a), the pump laser is focused on the target surface by objective 2 (Mitutoyo 10× M Plan APO Objective). The intermediate image is formed by objective 1 (Olympus PLN 10× Objective) and lens L1 (Lbtek, MAD420-A) and divided into two parts by a beam splitter (BS, Thorlabs, CCM1-BS013/M). A 400 nm bandpass filter (F1, Thorlabs, FBH400-10) together with some neutral density filters (Lbtek, NDFR-100C-4) is used to prevent the residual 800 nm component in the probe beam and the fluorescence generated during the ablation from entering the imaging systems. In the external imaging system, the *s*-polarized component is separated from the *p*-polarized component by a polarized beam splitter (PBS2, Lbtek, MPBS24-405) and the intermediate image is passed through lenses (L2-L3, Thorlabs, MAP1075150-A) into external cameras (CCD1-CCD2, Basler acA1920-40gm). The elements in Fig. 6(a) are the same as those in Fig. 5(a).

Supporting Note 2 **Simulation on the effects to the imaging quality with various spatial shift intervals between adjacent frames**

To verify the effects to the imaging quality of DI-CUP with various spatial shift intervals between adjacent frames through simulation, the same five dynamic scenes as those in Fig. 3 were selected, and each scene contains 15 time-varying images of size 256 × 256. In simulation, in constrast to the spatial shift interval between adjacent frames of 10 pixels in Fig. 3, the shift is tuned by 5, 15 and 20 pixels, respectively. The spatial encoding and spatiotemporal integration processes remain unchanged, and the PnP-FFDNet and PnP-TV-CD algorithms are still used for reconstruction. For each scene, the averaged PSNR (dB) and SSIM values are calculated, and the results obtained from the two algorithms are shown in Fig. S1. Obviously, both the PSNR and SSIM values increase as the spatial shift interval between adjacent frames increases, which is mainly contributed to the less overlap between them. Moreover, as the spatial structure complexity of the five scenes varies, the increase differs from each other apparently.

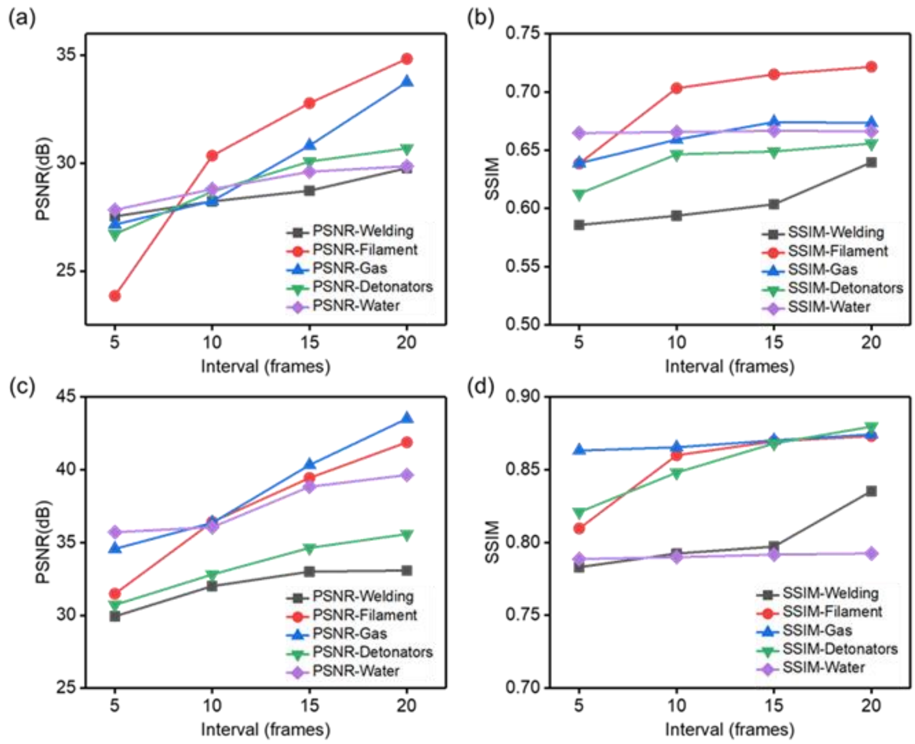

Figure S1 The averaged PSNR in dB and SSIM values of reconstructed images for five dynamic scenes with various spatial shift intervals between adjacent frames through (**a**) and (**b**) PnP-FFDNet, and (**c**) and (**d**) TV-CD.

Supporting Note 3 Simulation on the effects to the imaging quality with non-uniform intensities of sub-pulses

3.1 Results with intensity fluctuations

To verify the robustness of DI-CUP to intensity fluctuation among the sub-pulses, three of the scenes (Filament, Gas and Water) were selected for simulation. The odd numbered frames out of 15 images (8 images in total) were chosen and added with random intensity changes in the range of 0.8-1, and each scene has two different sets of random intensity values. Meanwhile, the spatial shift interval between adjacent frames changes from 5 to 20 pixels as it in Supporting Note 2. The calculated averaged PSNR and SSIM values of each reconstructed scene with the two kinds of intensity fluctuations and without intensity fluctuation obtained from both PnP-FFDNet and PnP-TV-CD are shown in Fig. S2, respectively. It can be seen that, with the fluctuation of intensity in consideration, the differences of each scene in PSNR calculated by the two algorithms are less than 3 dB, and the changes of SSIM are less than 0.02. Therefore, images can be reliably reconstructed within an intensity fluctuation of 20% in sub-pulses.

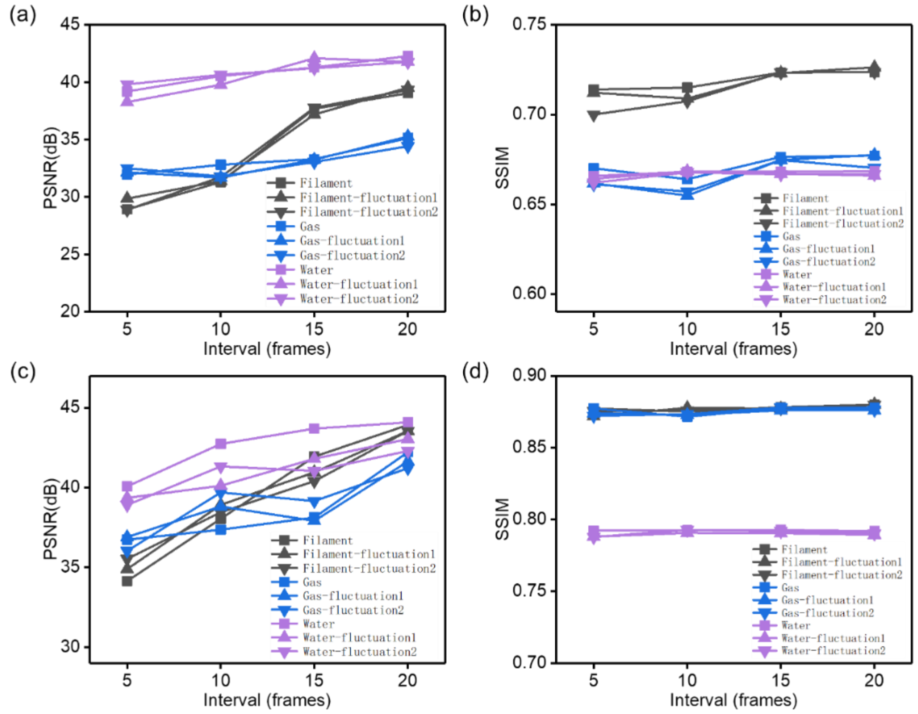

Figure S2 The averaged PSNR in dB and SSIM values of reconstructed images for three dynamic scenes with intensity fluctuations among sub-pulses through (**a**) and (**b**) PnP-FFDNet, and (**c**) and (**d**) TV-CD.

3.2 Results with intensity attenuation

To verify the robustness of DI-CUP to intensity attenuation among the sub-pulses, three of the scenes (Filament, Gas and Water) were selected for simulation. The odd numbered frames out of 15 images (8 images in total) were chosen and added with a sequential intensity attenuation ratio of 10% with respect to the previous one, resulting in the intensity of the last image being 48% of that of the first one under illumination. Similarly, the spatial shift interval between adjacent frames changes from 5 to 20 pixels as it in Supporting Note 2. The calculated averaged PSNR and SSIM values of each reconstructed scene with the intensity attenuation and with further intensity calibration obtained from both PnP-FFDNet and PnP-TV-CD are shown in Fig. S3, respectively. Here, the intensity calibration is conducted by renormalizing the images based on their illumination intensities accordingly. It can be seen that, although both of the metrics drop evidently with such a large intensity attenuation, they are primely recovered after the intensity calibration. Therefore, the intensity fluctuation should be pre-calibrated for further eliminating its effect on image reconstruction when the fluctuation cannot be ignored.

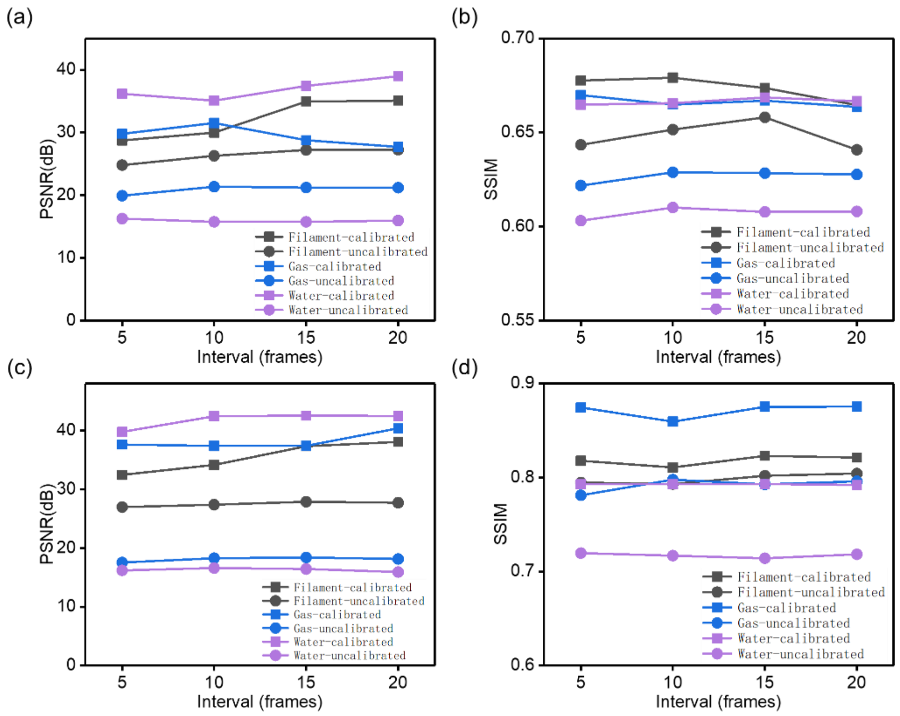

Figure S3 The averaged PSNR in dB and SSIM values of reconstructed images for three dynamic scenes with intensity attenuation and with further intensity calibration through (**a**) and (**b**) PnP-FFDNet, and (**c**) and (**d**) TV-CD.

Supporting Note 4 **1D streak measurement of pulse train generated by FP cavitiy**

In DI-CUP, the sub-pulse intensities of the pulse train provided by the FP cavity-based pulse shaper are decreasing, and the decay rate depends on the beam splitting ratio of BS2. The BS2 with a reflectivity to transmittance ratio (RTR) close to 9:1 at 0° incidence angle was customized to ensure the sub-pulse intensities decrease slowly. Figure S4(a) illustrates the intensity distribution image of different sub-pulses measured by the streak camera in 1D streak mode. It can be obviously seen that the intensities are decreasing gradually as expected. Moreover, the intensity along the x' direction of each sub-pulse exhibits a quasi-uniform distribution. For quantitative comparison, the distribution in Fig. S4(a) were integrated along the x' direction, and the normalized intensity profile is shown as the red solid line in Fig. S4(b). The reflectivity of the harmonic beam splitter (HBS) is close to 99%, and the theoretical values of the sub-pulse intensity attenuations (yellow rectangles) agree well with the measurments when the reflectivity of BS2 is 85%, indicating an actual RTR of 0.85:0.15. In image reconstruction, it is necessary to modify the intensities of the reconstructed frames based on the experimentally pre-calibrated sub-pulse energy distribution.

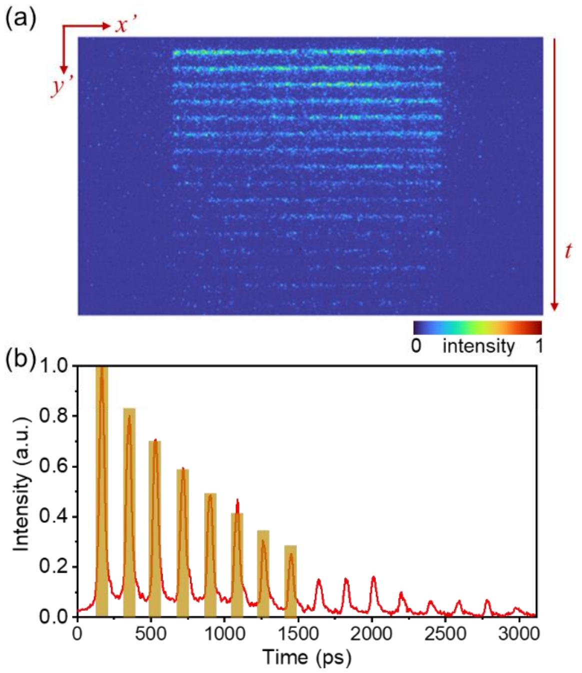

Figure S4 (**a**) Intensity distribution image of pulse train generated by an FP cavity with a beam splitting ratio of 9:1 obtained with 1D streak imaging; (**b**) The normalized intensity profile of the pulse train. Red solid line: Experimental measurements; Yellow rectangles: Theoretical calculations.
